# All-Bamboo Fiber Thermosetting Plastics with Excellent Mechanical Properties, Degradability and High Water Resistance

**DOI:** 10.3390/polym18020220

**Published:** 2026-01-14

**Authors:** Wenjun Zhang, Wenting Ren, Enbo Liu, Chunyan Mou, Jiawei Han, Jing Lv, Dengkang Guo

**Affiliations:** 1 College of New Energy and Materials, China University of Petroleum (Beijing), Beijing 102249, China; zwj2019010584@163.com; 2 Engineering Technology Research Center for Building and Decorating Materials of Bamboo State Forestry Administration, China National Bamboo Research Center, Hangzhou 310012, China; javierh@163.com; 3 Zhejiang Academy of Forestry, Hangzhou 310023, China; 18856022706@163.com; 4 Luzhou Academy of Forestry, Luzhou 646000, China; 13541497030@163.com (E.L.); 13551727165@163.com (C.M.)

**Keywords:** bamboo fiber, thermosetting plastics, mechanical properties, degradability, water resistance

## Abstract

Petroleum-based plastics are non-renewable and degrade poorly, persisting in the environment and causing serious ecological pollution, so urgent development of alternatives is needed. In this study, all-bamboo fiber thermosetting plastics (BTPs) were successfully prepared through selective sodium periodate oxidation of bamboo fibers followed by hot-pressing. The results demonstrate that the oxidation treatment effectively enhanced fiber reactivity and facilitated the formation of dense composite materials during hot-pressing. Compared with petroleum-based plastics (e.g., PVC), BTPs exhibit outstanding mechanical properties: flexural strength reaches 100.73 MPa, tensile strength reaches 83.31 MPa, while the 72 h water absorption and thickness swelling rates are as low as 5.36% and 4.59%, respectively. This study also reveals the mechanism by which residual lignin affects material microstructure formation through competitive oxidation reactions. Although it imparts initial hydrophobicity, it hinders complete fiber activation, leading to the formation of micro-defects. Furthermore, BTPs can completely degrade in 1% NaOH solution within 24 h, demonstrating excellent degradability. This research provides a new strategy for developing high-performance, degradable all-bamboo-based materials and promotes the value-added utilization of bamboo resources.

## 1. Introduction

Petroleum-based plastics, as a class of polymeric materials, have achieved global ubiquity owing to their low cost, lightweight nature, ease of large-scale manufacturing, and generally remarkable durability [1]. However, the durability from their high molecular weight, hydrophobicity, and lack of functional groups susceptible to microbial enzymatic degradation [2,3] inherent traits that render plastics highly persistent in natural environments. Their accumulation has resulted in severe contamination of soil and water, exerts significant impacts on climate change, and poses potential risks to human health [3]. Plastic pollution now stands as an urgent global environmental challenge, with its adverse effects on ecosystems and human health becoming increasingly evident. In response to this crisis, and driven by the dual imperatives of technological advancement and sustainability, scientific attention has increasingly turned toward towards natural materials. Consequently, a variety of biomass-derived materials, such as wood [4], chitosan [5], bamboo [6], mussel proteins [7], and algae [8] have been engineered into biodegradable and environmentally benign bioplastics.

Bamboo is recognized for its excellent toughness, high mechanical strength, good wear resistance, and dense microstructure [9]. Compared to wood, bamboo has a shorter growth cycle, only 3–4 years [10], lower cost, and a wider range of sources. Owing to these advantages, bamboo is regarded as a highly promising biomass resource. Driven by the “Bamboo as a Substitute for Plastics” initiative and global plastic restriction policies, bamboo demonstrates significant advantages over conventional plastics in terms of resource efficiency, environmental impact mitigation, and ecological benefits [11]. In recent years, researchers have successfully developed numerous bamboo-based composites as sustainable alternatives to plastics. Chen et al. developed a high-utility bamboo fiber tableware product by employing a novel hybrid fiber-membrane strategy. Using this material, 20% of plastic tableware can be replaced, equivalent to reducing the carbon emissions of 300,000 cars in a small city [12]. Han et al. transformed natural bamboo into a lightweight, high-strength bamboo-based material by removing lignin and hemicellulose from the bamboo surface, followed by in situ infiltration and curing of phenolic formaldehyde resin for densification. This material shows promise as a sustainable green alternative to non-renewable synthetic materials [13]. Lan et al. developed a thermosetting bamboo-based plastic alternative by integrating bamboo powder (BP) into a dynamic network of polyimide (PI) through hot-pressing. The BP/PI system demonstrated excellent mechanical properties due to the formation of multiple hydrogen bonding interactions between BP and PI at the interface, achieving superior interfacial compatibility [14]. However, these materials still present several challenges. For instance, all-bamboo materials exhibit relatively poor mechanical properties, while the incorporation of petroleum-based polymers results in unsatisfactory degradation performance [15]. Moreover, inherent incompatibility between hydrophilic plant fibers and hydrophobic polymer matrices leads to weak interfacial adhesion and inefficient stress transfer [16].

Furthermore, industrial-scale production of bamboo fiber (BF) is now commercially available. BF is a natural fiber extracted from bamboo through chemical treatment, and can be produced via chemical pulping methods that are not strictly limited by the species, source, or quality of bamboo. This flexibility enables the utilization of bamboo waste for BF extraction [17]. Furthermore, as most lignin in bamboo is removed during the chemical pulping process [18], the direct use of bamboo fibers eliminates the need for additional delignification steps, offering advantages in both environmental sustainability and economic efficiency.

The direct use of BF faces challenges such as strong hydrophilicity, weak interfacial adhesion, and low reactivity, which hinder its plastic molding. However, previous studies have demonstrated that sodium periodate oxidation can markedly enhance both the plasticity and reactivity of BF [19,20]. This oxidative reaction specifically cleaves the C2–C3 bonds in cellulose chains and converts diol into dialdehyde [21], thereby markedly improving the chemical reactivity of BF. The treated fibers exhibit processing characteristics comparable to those of petroleum-based polymers and can be thermoformed into complex all-bamboo-based structures [20]. Consequently, BF represents an ideal raw material for bamboo-based alternatives to plastics [22].

Based on the aforementioned background, this study successfully fabricated all-bamboo fiber thermosetting plastics (BTPs). First, selective oxidation of BF with sodium periodate was employed to modulate its chemical properties [23], yielding activated bamboo fiber (BAF) that is highly reactive and readily thermoformable. To further investigate the role of lignin during the oxidation process, BF was subjected to bleaching treatment [24] to prepare fully delignified bamboo cellulose fiber (BCF) [25]. The resulting BCF was then oxidized under the same conditions to obtain activated bamboo cellulose fiber (BCAF), enabling a systematic evaluation of lignin’s influence on the oxidation process. Because the aldehyde groups in BAF and BCAF primarily exist in the form of hemiacetals and hydrated dialdehydes in the dry state [26], their flowability and moldability during hot-pressing are compromised because some aldehydes further react with hydroxyl groups to form a three-dimensional network. To address this, based on the activation mechanism of water molecules toward aldehyde [27], the activated fiber samples were first ground and moistened, followed by hot-press molding of BAF and BCAF at 90 °C and 20 MPa, respectively. During this process, water molecules facilitate the dissociation of aldehyde from their condensed forms, thereby promoting intra- and intermolecular crosslinking of aldehyde, forming acetal and hemiacetal bonds [28]. This effectively enhances the interfacial adhesion, leading to markedly improved mechanical properties and water resistance of the material [29]. After cooling and setting, the BTPs were successfully obtained. The product hot-pressed from BAF was designated as activated bamboo fiber thermosetting plastic (BAFTP), while that from BCAF was named activated bamboo cellulose fiber thermosetting plastic (BCAFTP).

As a type of all-bamboo-based plastics, BTPs demonstrate outstanding environmental compatibility. These characteristics make BTPs an ideal sustainable material that shows promise for replacing conventional petroleum-based plastics and bamboo–plastic composites in various application scenarios. Owing to their remarkable moldability, high strength, and water resistance, these materials hold potential for the customized design and manufacturing of complex-shaped components in the future. Furthermore, the all-biomass plastics strategy proposed in this study can be extended to the value-added conversion of cellulose-rich biomass wastes such as waste paper and express packaging materials [30]. This approach would not only help alleviate environmental pressures but also generate substantial economic benefits.

## 2. Materials and Methods

### 2.1. Materials

The bamboo fiber (BF) was collected from a Sichuan Jinzhu Paper Industry Co., Ltd., Luzhou, Sichuan, China. Isopropyl alcohol and sodium periodate of analytical grade, acquired from Aladdin Co., Ltd., Shanghai, China. were employed directly in their as-purchased form without any additional purification processes.

### 2.2. Sample Preparation

Bamboo cellulose fiber (BCF) was obtained by bleaching BF. Subsequently, 55 g of BF and 55 g of BCF were separately dispersed in 1600 mL of deionized water for 2 h. Then, 106 g of sodium periodate and 50 mL of isopropanol were added to each mixture [19,20]. The reactions were conducted at 50 °C in the dark for 6 h. Subsequently, the samples were filtered. After samples were thoroughly rinsed with distilled water, they were dried at 30 °C to obtain activated fibers, designated as BAF and BCAF. Finally, each activated fiber sample (adjusted to 40% water content) was hot-pressed at 95 °C and 20 MPa for 5 min to prepare the all-bamboo fiber thermosetting plastics (BTPs), correspondingly designated as BAFTP and BCAFTP.

### 2.3. Chemical Component Analysis

The lignin of samples was analyzed using the Laboratory Analytical Procedure (LAP). Initially, the samples underwent extraction with a benzene–alcohol mixture (2:1 ratio). This was followed by hydrolysis using 72% sulfuric acid at 30 °C for 1 h in a water bath. Subsequently, the mixture was autoclaved with 4% sulfuric acid at 121 °C for 1 h (Yxq-Ls-50SII, Shanghai, China). The solution was filtered, and the residue was washed. The washed residue is then weighed and subsequently calcined in a Muffle furnace (Nabertherm N7/H, Hohenbrunn, Germany) for determination of lignin content.

The chemical composition of the samples, including cellulose and hemicelluloses, were quantified by the Chinese standards (GB/T 2677.8-2021 [31] and GB/T 2677.10-2022 [32], respectively).

### 2.4. Aldehyde Content Determination

The procedure involves taking 0.15 g of the sample and reacting it with 25 mL 0.25 M (pH 4.0) hydroxylamine hydrochloride solution at room temperature for 4 h. After the reaction, 0.1 M NaOH titrated the solution back to pH 4.0. The aldehyde content (AC) was determined by titration with hydroxylamine. The aldehydes react with hydroxylamine hydrochloride to form HCl, which is then titrated with NaOH.

AC was calculated from NaOH used:

AC = V_NaOH_ × C_NaOH_/m_0_

where V_NaOH_ represents the volume of NaOH used, and C_NaOH_ represents the concentration of NaOH used, and m_0_ is the mass of the sample. Each titration experiment is independently repeated three times, and the results are averaged.

### 2.5. Mechanical Test

The mechanical properties tests were conducted by using a universal tensile testing machine (Electromechanical Universal Testing Machine, Maximum load: 1 kN, Shenzhen Xinsansi Material Testing Co., Ltd., Shenzhen, China). The tensile strength of the samples was evaluated according to the standard GB/T 1040.2-2022 [33]. The samples were processed into a dumbbell-shape specimen and stretched at a deformation rate of 2 mm/min over a 20 mm span. For the flexural strength, the samples, measuring 40 × 10 × 1 mm^3^, are tested at a deformation rate of 2 mm/min over a 30 mm span.

Each experiment was performed in six independent replicates. The mean value and standard deviation were used for plotting. Statistical analysis and visualization were performed using Origin. A maximum single deviation of the data from the mean within 3% is considered acceptable.

### 2.6. Water Resistance Analysis

The water contact angle of the sample was measured by DSA 30 contact angle meter (KRUSS, Hamburg, Germany).

Absolute dry samples measuring 10 × 10 × 3 mm^3^ were submerged in a container with 10 cm of water to evaluate their water resistance. The water absorption (WA) rate and thickness swelling (TS) rate of the samples were calculated using the following formulas:

WA = (M_2_ − M_1_)/M_1_ × 100

TS = (H_2_ − H_1_)/H_1_ × 100

where M_1_ represents the absolute dry mass of the sample, M_2_ represents the mass of the sample after water immersion, H_1_ represents the absolute dry thickness of the sample, and H_2_ represents the thickness of the sample following water saturation.

Each experiment was performed in six independent replicates. The mean value and standard deviation were used for plotting. Statistical analysis and visualization were performed using Origin. A maximum single deviation of the data from the mean within 3% is considered acceptable.

### 2.7. Degradability Test

Rectangular samples measuring 40 × 5 × 1 mm^3^ were immersed in containers with 1% NaOH solution and observed regularly over a set period of time to assess their chemical degradability.

### 2.8. Characterizations

The chemical structural changes in sample were investigated by utilizing Fourier Transform Infrared Spectroscopy (FTIR, PerkinElmer Spotlight 400, PerkinElmer, Shelton, CT, USA). The crystalline structure changes in cellulose fiber were analyzed through X-ray Diffraction (XRD, D8, Bruker, Karlsruhe, Germany). Scanning Electron Microscopy (SEM, Regulus 8100, Tokyo, Japan) was used to further observe the cross-sectional morphology and analyzed the microstructural changes in the samples. Thermogravimetric analysis (TGA, STA 449F3, Netzsch-Gerätebau GmbH: Selb, Germany) under an N_2_ atmosphere. The temperature was raised from room temperature to 600 °C at a heating rate of 10 °C/min using a sample mass of 5–8 mg.

## 3. Results and Discussion

### 3.1. Bamboo Fiber Chemical Modification

**Figure 1:**
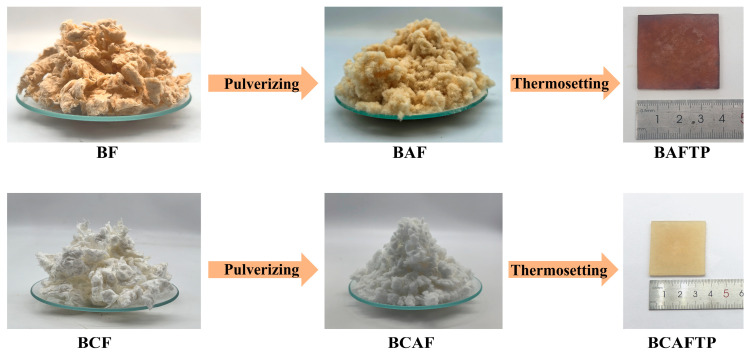
Schematic diagram of BTPs preparation.

**Figure 2:**
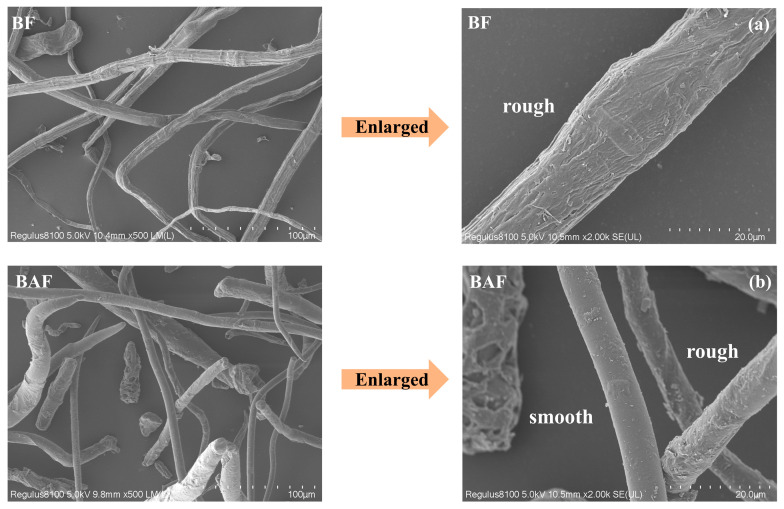
SEM photo of BF and BAF. (**a**) SEM photo of BF; (**b**) SEM photo of BAF.

**Figure 3:**
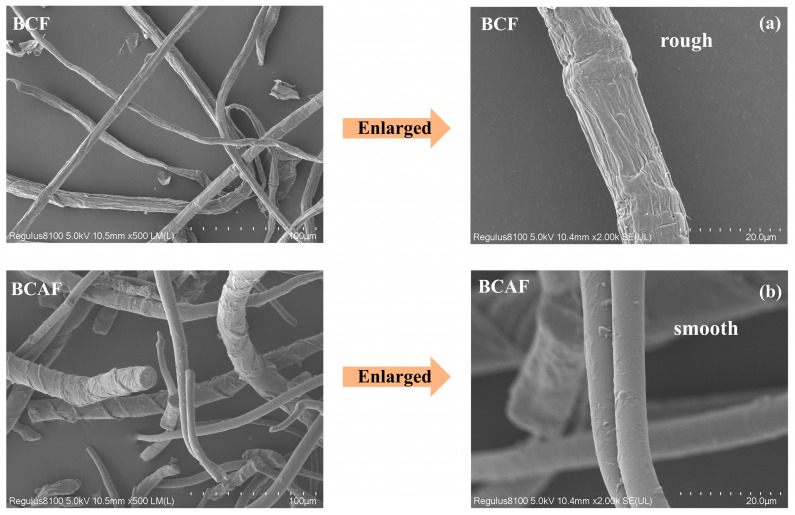
SEM photo of BCF and BCAF. (**a**) SEM photo of BCF; (**b**) SEM photo of BCAF.

As shown in Figure 1, BF exhibited a brown color, whereas BCF appeared white. Chemical quantitative analysis revealed that this color difference is attributed to the residual lignin content (1.67%) in BF (Table S1). SEM images (Figure 2a and Figure 3a) revealed that both BF and BCF displayed rough surface morphologies. This roughness results from the substantial removal of lignin during the chemical pulping process, which induces the separation of lignin and hemicellulose, leading to the exposure and aggregation of cellulose fibers [34,35]. The similar morphologies of BF and BCF indicate that the bleaching process did not alter the morphology of fiber. Measurements showed that BF had an average length of 1.25 mm, a diameter of 52.27 μm, and an aspect ratio of 23.91 (Table S2). In contrast, BCF had an average length of 1.33 mm, a diameter of 52.76 μm, and an aspect ratio of 25.21 (Table S3). Both BF and BCF retain the characteristic high-aspect-ratio structure of bamboo fiber cells [17,36], demonstrating that the chemical pulping process did not disrupt their fibrous architecture [37].

A comparative analysis of Figure 2a,b reveals that BF exhibited a rough surface morphology, whereas the BAF obtained after sodium periodate oxidation displays a smoother surface with attached particulate matter. The measured lengths of BF (1.25 mm) and BAF (1.39 mm) remained largely unchanged; however, the diameter of BAF (22.75 μm) was markedly smaller than that of BF (52.27 μm) (Tables S2 and S4). Similarly, comparison of Figure 3a,b shows that BCAF becomes notably smoother compared to BCF. The lengths of BCF (1.33 mm) and BCAF (1.47 mm) show little variation, while the diameter of BCAF (20.02 μm) was markedly reduced relative to BCF (52.76 μm) (Tables S3 and S5). These morphological changes can be attributed to the collapse of parenchyma cell walls and the formation of smoother cell walls following sodium periodate treatment, accompanied by a reduction in cell lumen volume [38]. Consequently, the fibers appear thinner and exhibit smoother surfaces under SEM observation.

It is noteworthy that a comparison of Figure 2b and Figure 3b reveals distinct differences between BAF and BCAF. Although both fibers exhibit relatively smooth morphologies, BAF displays obvious particulate matter on its surface and has a larger diameter than BCAF. In light of previous studies, these observations are attributed to the residual lignin in BAF, which competes with cellulose for reaction with sodium periodate during the oxidation process, thereby impeding the effective oxidation of the fibers [19,39].

**Figure 4:**
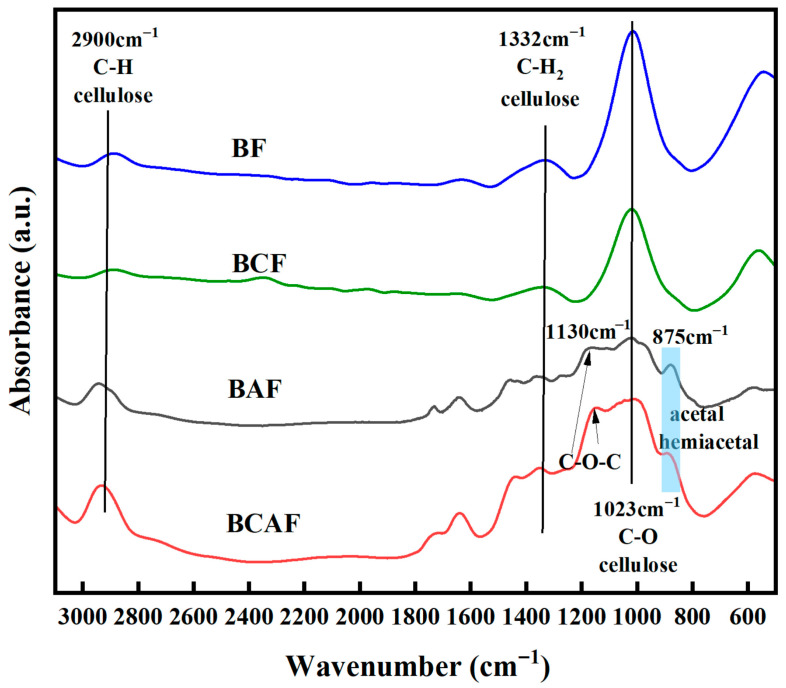
FTIR of BF, BCF, BAF and BCAF.

FTIR analysis revealed the chemical structural changes in BF and BCF following sodium periodate oxidation. As shown in Figure 4, the spectra of all samples display absorption peaks about 2900 cm^−1^, 1332 cm^−1^ and 1023 cm^−1^, corresponding to the cellulose [34]. In both BAF and BCAF curves, an absorption band appears at 875 cm^−1^, corresponding to the hydrated structure of acetal and hemiacetal [18]. This is attributed to the aldehydes generated from the sodium periodate oxidation of cellulose, these aldehydes undergo condensation with adjacent hydroxyl groups under dry conditions, forming acetal and hemiacetal hydrate structures [40,41].

**Figure 5:**
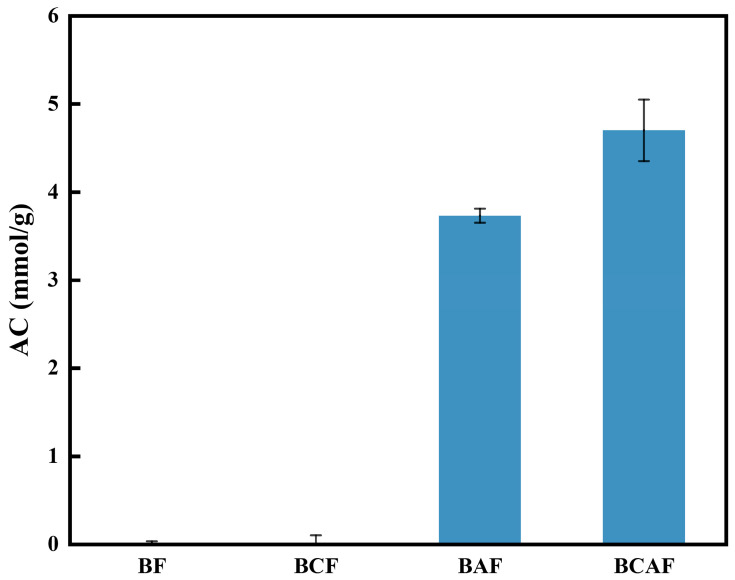
AC test of BF, BCF, BAF, BCAF.

The degree of aldehyde functionalization was further evaluated by quantitatively determining the aldehyde content (AC) of the samples using an oxidation titration method. As shown in Figure 5, both BF and BCF exhibited very low aldehyde contents, approaching 0 mmol/g. In contrast, after oxidation with sodium periodate, the aldehyde contents of BAF and BCAF increased significantly, reaching 3.73 mmol/g and 4.70 mmol/g, respectively. Notably, the aldehyde content of BAF was considerably lower than that of BCAF. This finding further confirms that the presence of lignin competitively consumes sodium periodate, thereby inhibiting its effective oxidation of the sugar units in cellulose.

### 3.2. Preparation of BTPs

**Figure 6:**
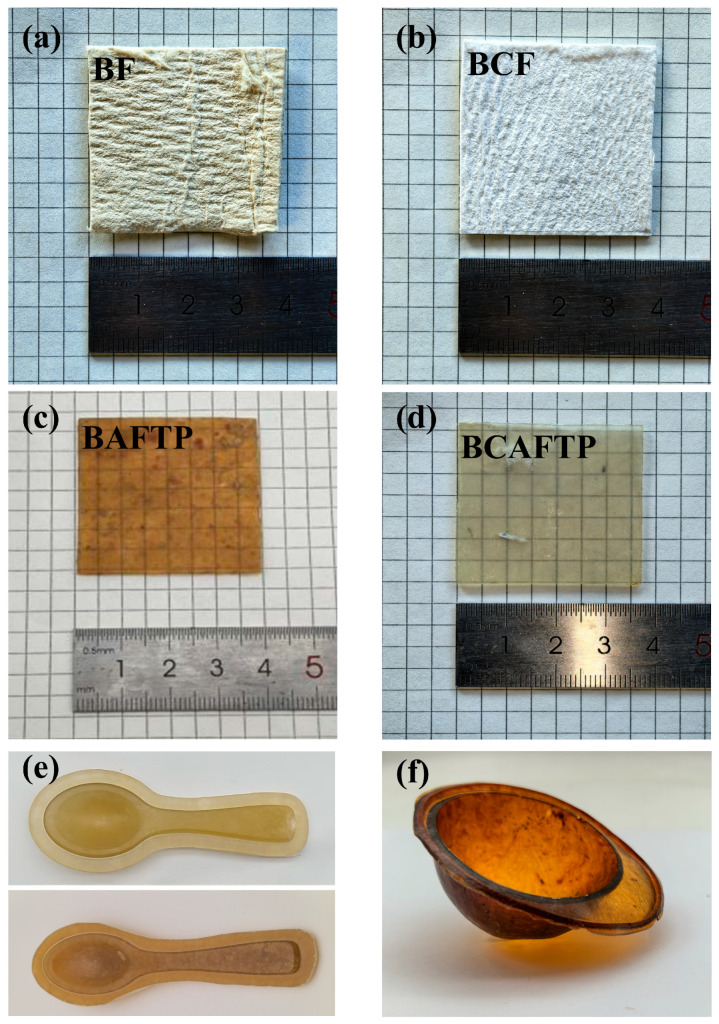
Sample photo of BTPs. (**a**) Sample photo of direct pressing of BF; (**b**) Sample photo of direct pressing of BCF; (**c**) Sample photo of BAFTP; (**d**) Sample photo of BCAFTP; (**e**,**f**) Sample photos of BTPs with different shapes.

Direct pressing of BF and BCF produced bamboo pulp boards, as shown in Figure 6a,b. However, these materials are opaque, lack plastic-like gloss, and exhibit a cardboard-like texture. In contrast, the BAFTP and BCAFTP materials prepared from BAF and BCAF via thermosetting (Figure 6c,d) demonstrate transparency and a plastic-like appearance. This difference stems from the strong intermolecular hydrogen bonding in native cellulose, which restricts the chain mobility and processability of the fibers [42]. After treatment with sodium periodate, the hydroxyl groups on the cellulose chains are converted to aldehyde. This transformation not only loosens the originally dense cellulose microfibril structure but also reduces its crystallinity (Figure S1), thereby markedly enhancing the reactivity and flow characteristics of the fibers [43] and enabling their plastic molding through thermosetting [44].

A comparison of Figure 6c,d reveals that BAFTP contains distinct flocculent heterogeneous structures, whereas BCAFTP is macroscopically uniform. This phenomenon is primarily attributed to the residual lignin in BAF, which competitively consumes sodium periodate during the oxidation process. As a result, a portion of the fibers remains insufficiently activated, thereby hindering complete densification of the material during hot-pressing.

Furthermore, a comparison between Figure 6a,c shows that the color of BAFTP darkens markedly after thermosetting. This darkening can be attributed to two main reasons. First, during the hot-pressing process, the unsubstituted reactive hydrogen atoms on the lignin benzene rings underwent hydroxymethylation and polycondensation reactions with an aldehyde, generating dark-colored products [45,46]. Second, during the oxidation process, sodium periodate was used to oxidize the hydroxyl present in the interunit linkages (β-O-4 bond) in the lignin structure and partly convert lignin to quinones [47].

**Figure 7:**
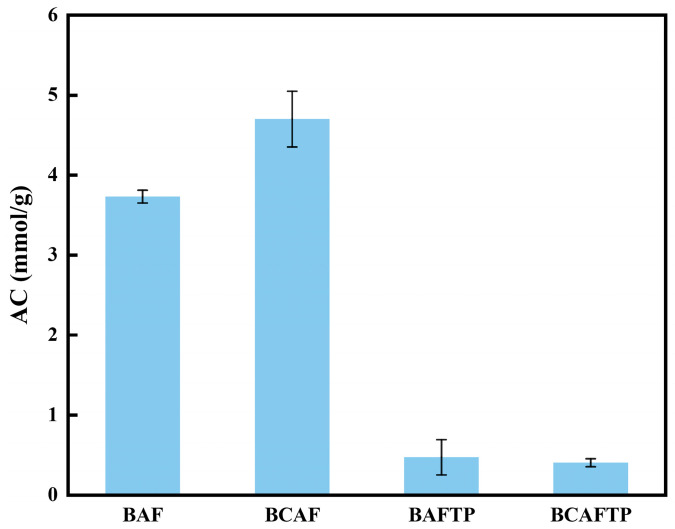
AC values of BAF, BCAF, BAFTP and BCAFTP.

The AC test results presented in Figure 7 elucidate the bonding mechanism between fibers in the BTPs samples. As shown in Figure 7, the oxidized bamboo fibers are rich in reactive aldehyde groups. After hot-pressing, the AC values of BAFTP and BCAFTP decreased to 0.47 mmol/g and 0.40 mmol/g, respectively, which are markedly lower than those of the pre-treated BAF and BCAF. This reduction indicates that aldol condensation reactions occurred between the aldehyde groups and hydroxyl groups in the fibers during the hot-pressing process. It is a description of the cross-linking mechanism involved in forming the thermosetting network. It is noteworthy that the more pronounced decrease in AC value for BCAFTP suggests that a more extensive acetal cross-linking reaction took place during its hot-pressing formation, resulting in a denser polymeric network structure [48]. This finding provides key evidence for explaining the differences in macroscopic properties between BAFTP and BCAFTP.

The preparation process of BTPs is highly intriguing. During hot-pressing, the aldehyde and hydroxyl groups on the activated fibers undergo an aldol condensation reaction, forming a strong covalent bond network that transforms the material from thermoplastic to thermosetting.

### 3.3. Mechanical Properties of BTPs

**Figure 8:**
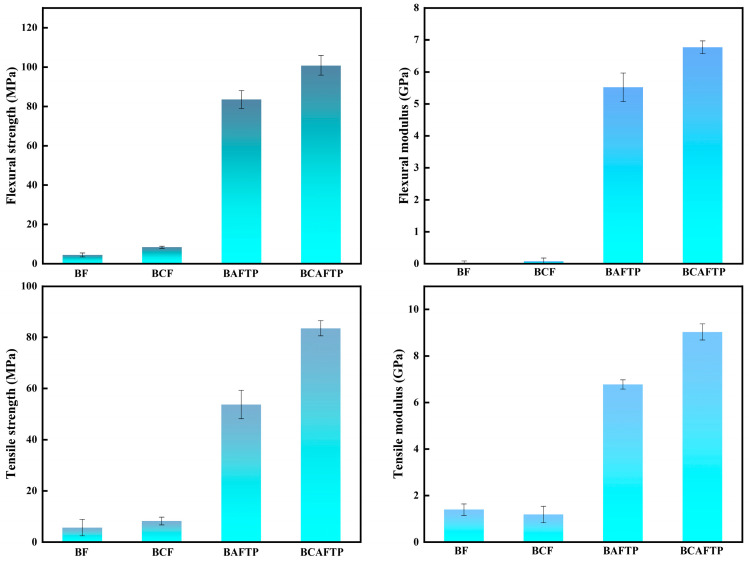
Comparison of Mechanical Properties of BTPs.

To investigate the potential applications of BTPs, mechanical property evaluations were carried out focusing on two critical parameters: flexural and tensile performance. As shown in Figure 8, panels directly pressed from BF and BCF exhibited poor mechanical properties. Specifically, BF had a flexural strength and modulus of 4.40 MPa and 0.02 GPa, respectively, and tensile strength and modulus of 5.62 MPa and 1.39 GPa, respectively. BCF displays slightly superior mechanical properties compared to BF, with flexural strength and modulus reaching 8.30 MPa and 0.08 GPa, respectively, and tensile strength and modulus achieving 8.16 MPa and 1.18 GPa, respectively. The mechanical properties of the boards directly produced from BF and BCF were slightly higher than those reported by Liu et al., and the mechanical strength of food packaging produced from recycled fiber molded pulp (SFMP) [49]. Therefore, BF and BCF direct-pressed panels are generally suitable only for single-use products, owing to their inferior mechanical performance.

After hot-pressing, BAFTP exhibited a remarkable improvement in mechanical properties, achieving a flexural strength of 83.44 MPa and a modulus of 5.52 GPa. These values represent increases by factors of 18.96 and 276 in flexural strength and modulus, respectively, over those of BF panels. Meanwhile, the tensile strength and modulus of BAFTP were 53.74 MPa and 6.77 GPa, corresponding to 9.56-fold and 4.87-fold improvements over the directly pressed BF panels. Similarly, BCAFTP also showed markedly enhanced mechanical performance after hot-pressing, with flexural strength and modulus reaching 100.73 MPa and 6.77 GPa, respectively. These values are 12.14 and 84.63 times higher than those of BCF panels. Furthermore, BCAFTP attained a tensile strength of 83.31 MPa and a modulus of 9.03 GPa, indicating 10.21-fold and 7.65-fold enhancements compared to the directly pressed BCF panels.

The ANOVA results (Table S8) revealed that BCAFTP demonstrates superior mechanical performance compared to BAFTP and all-bamboo-based thermosetting plastics (ABTPs) [20]. Specifically, its flexural strength is 1.21 times that of BAFTP (83.44 MPa), and 1.26 times that of ABTPs (approximately 80 MPa) [20]. Similarly, its tensile strength reaches 1.49 times that of BAFTP (53.74 MPa), and 1.67 times that of ABTPs (approximately 50 MPa) [20]. In terms of modulus, BCAFTP exhibited 1.23 times the flexural modulus and 1.33 times the tensile modulus of BAFTP.

Furthermore, PVC is one of the most widely used conventional petroleum-based plastics globally. It is extensively applied in construction materials, daily necessities, and packaging. With its high strength, water resistance, and low cost, it stands as a prime example of traditional non-biodegradable plastics. The fact that BTPs meet or exceed PVC’s key performance metrics, such as tensile strength and flexural strength, demonstrates their practical viability as an alternative to petroleum-based plastics. Surprisingly, the flexural strength of BCAFTP is 1.46 times that of PVC (68.95 MPa) [12], Similarly, its tensile strength reaches 1.55 times that of PVC (51.71 MPa) [12]. In conclusion, BCAFTP can serve as an all-bamboo-based plastic alternative to conventional petroleum-based plastics such as PVC.

**Figure 9:**
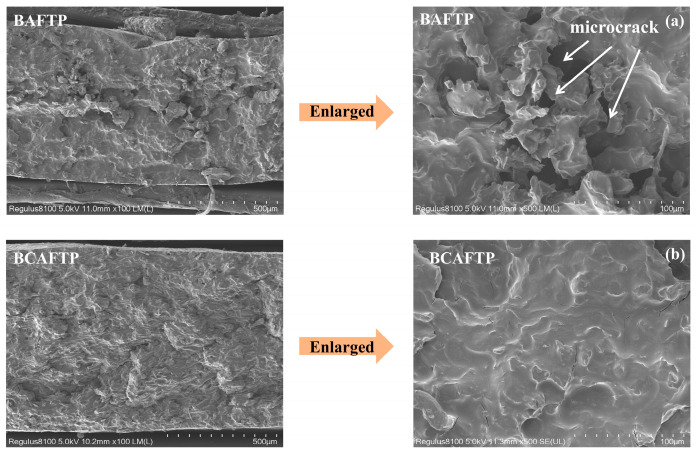
SEM photo of BAFTP and BCAFTP. (**a**) SEM photo of BAFTP; (**b**) SEM photo of BCAFTP.

Data analysis and literature reports indicate that residual lignin also affects the mechanical properties of BTPs [50,51]. To further elucidate the differences in mechanical performance between BAFTP and BCAFTP, their cross-sections were examined by SEM. As shown in Figure 9a, BAFTP contains microcracks and agglomerates. This morphology results from hindered fiber activation due to residual lignin, which limits fiber flow and bonding during hot-pressing. In contrast, the cross-section of BCAFTP appears dense and uniform, with no significant defects, cracks, or agglomeration observed, further explaining why BAFTP exhibited inferior macroscopic mechanical properties compared to BCAFTP.

### 3.4. Water Resistance of BTPs

**Figure 10:**
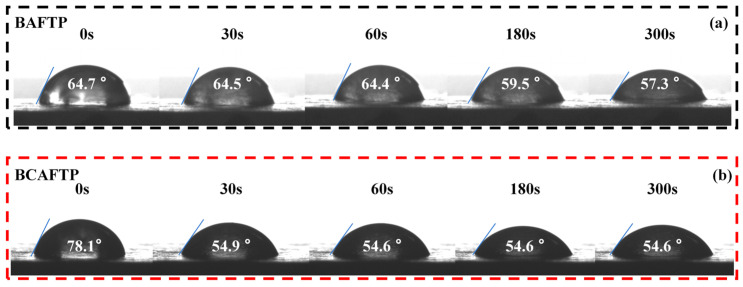
Water contact angle of BTPs. (**a**) water contact angle of BAFTP; (**b**) water contact angle of BCAFTP.

The inherent hydrophilicity of bamboo markedly compromises the water resistance of bamboo-based materials [52]. To systematically evaluate the durability of BTPs in humid environments, this study conducted contact angle measurements and water resistance analysis. As shown in Figure 10, the contact angles of both BAFTP and BCAFTP decrease over time due to the intrinsic hydrophilic nature of bamboo. However, compared with untreated bamboo powder boards (Figure S8), the contact angles of both BTPs decrease at a markedly slower rate, indicating markedly improved water resistance. This enhancement is primarily attributed to material densification during hot-pressing and the stable chemical bonding formed between activated bamboo units. Notably, BCAFTP exhibits a slightly lower initial contact angle than BAFTP. This may be due to the hydrophobic contribution of residual lignin in BAFTP [53], whereas the more thorough lignin removal during the bleaching process of BCF results in relatively higher hydrophilicity for BCAFTP. Nevertheless, at 300 s, the contact angles of BAFTP and BCAFTP become essentially identical. This convergence could be attributed to the hydrophilicity of incompletely activated fibers in BAFTP or to water penetration into the microcracks within its structure.

**Figure 11:**
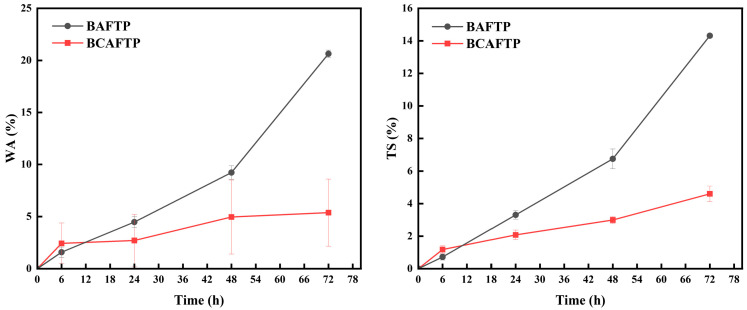
WA and TS of BTPs.

To further investigate the long-term water resistance of BTPs, water immersion tests were conducted. As shown in Figure 11, the water absorption (WA) and thickness swelling (TS) results further confirm the enhanced water resistance of BTPs. During the initial 6 h of immersion, both the WA and TS of BAFTP were lower than those of BCAFTP. This initial advantage is primarily attributed to the hydrophobic nature of the residual lignin in BAFTP. However, with prolonged immersion time, the WA and TS of BAFTP increased markedly, surpassing those of BCAFTP. This trend is attributed to the lignin hindering fiber oxidation, which led to incomplete activation of some fibers. The resulting stacked structures and voids within BAFTP created pathways for water penetration. In contrast, BCAFTP maintained relatively low WA and TS throughout the test, reaching only 5.36% and 4.59%, respectively, after 72 h of immersion-substantially lower than those of natural bamboo [54]. This excellent performance stems from the dense, chemically bonded network formed during the hot-pressing of fully activated fibers, which markedly improved the material’s water resistance.

### 3.5. Alkaline Degradation of BTPs

**Figure 12:**
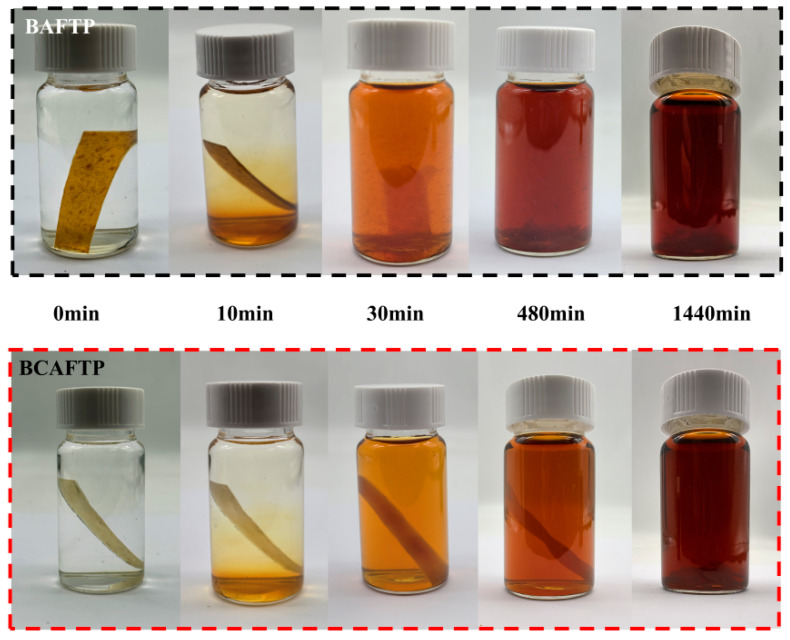
Alkaline Degradation of BTPs.

BTPs exhibit not only excellent water resistance but also notable degradability. As shown in Figure 12, the dissolution behavior of BTPs in a 1% NaOH solution was evaluated at room temperature. The experiments revealed that the samples began to degrade within 10 min of immersion in the alkaline solution, showing signs of phase separation, although the overall morphology of both BAFTP and BCAFTP remained largely intact at this stage. After 30 min of immersion, BAFTP started to break apart and disintegrate, accompanied by a darkening of the solution color. In contrast, BCAFTP maintained a relatively intact structure with only some substances dissolving from the surface and the solution gradually darkening. By 8 h, BAFTP had completely lost its original form, with flocculent degradation products settling at the bottom and the solution turning reddish-brown. Although BCAFTP retained a more complete overall shape, obvious bulging and fragmentation were observed on its surface, and the solution color was lighter than that of the BAFTP system. After 24 h of treatment, both samples were fully degraded, resulting in a dark brown homogeneous solution containing the dissolved products.

This controlled degradation behavior arises because sodium periodate oxidation selectively cleaves the glucopyranose rings within cellulose and hemicellulose. The remaining β-1,4-glycosidic bonds are then broken in alkaline conditions, resulting in the disintegration of the material [55]. These findings demonstrate that BTPs degrade rapidly and completely in alkaline environments, indicating their excellent degradability.

## 4. Conclusions

In this study, all-bamboo fiber thermosetting plastics (BTPs) were successfully prepared through selective sodium periodate oxidation of bamboo fibers followed by hot-pressing. BTPs demonstrates outstanding mechanical performance. The maximum flexural strength and modulus reach 100.73 MPa and 6.77 GPa, the maximum tensile strength and modulus achieve 83.31 MPa and 9.03 GPa, markedly exceeding those of conventional petroleum-based plastics such as PVC. Furthermore, BTPs exhibit excellent water resistance, with 72 h WA and TS as low as 5.36% and 4.59%, substantially lower than those of natural bamboo. Notably, BTPs completely dissolves completely in a 1% NaOH solution within 24 h, indicating favorable degradability. BTPs possess significant application potential within the strategic framework of “Bamboo as a Substitute-for Plastics,” providing a sustainable technological pathway for the high-value utilization of bamboo resources.

## Data Availability

The original contributions presented in this study are included in the article/Supplementary Material. Further inquiries can be directed to the corresponding authors.

## Supplementary Materials

The following supporting information can be downloaded at: https://www.mdpi.com/article/10.3390/polym18020220/s1, Figure S1: XRD of BCAF and BCF; Figure S2: TGA and DTG of BCAFTP and BAFTP; Figure S3: High temperature stability test (90 °C); Figure S4: High temperature stability test (120 °C); Figure S5: High temperature stability test (160 °C); Figure S6: Comparison of flexural curves; Figure S7: Comparison of tensile curves; Figure S8: Contact angle of BF; Figure S9: Inadequate hot pressing results in unformed samples; Table S1: Comparison of lignin content between BF and BCF; Table S2: Statistical analysis of BF dimensions; Table S3: Statistical analysis of BCF dimensions; Table S4: Statistical analysis of BAF dimensions; Table S5: Statistical analysis of BCAF dimensions; Table S6: Single factor ANOVA analysis of flexural properties; Table S7: Single factor ANOVA analysis of tensile properties; Table S8: Tukey HSD multiple comparisons results of mechanical properties; Table S9: BTPs’ two factor ANOVA analysis of WA and TS.
