# Supplementary material for: All-Bamboo Fiber Thermosetting Plastics with Excellent Mechanical Properties, Degradability and High Water Resistance
Source: Polymers (Basel). 2026 Jan 14;18(2):220. doi: 10.3390/polym18020220 (PMC12845541; doi:10.3390/polym18020220)
Supplement: Supplementary file 1 [file polymers-18-00220-s001.zip › polymers-4062584-supplementary.pdf]

All-Bamboo Fiber Thermosetting Plastics with Excellent Mechanical Properties, Degradability and High Water Resistance

Wenjun Zhang [1,2], Wenting Ren [3], Enbo Liu [4], Chunyan Mou [4], Jiawei Han [2], Jing Lv [1,*] and Dengkang Guo [2,*]

[1] College of New Energy and Materials, China University of Petroleum (Beijing), Beijing 102249, China
[2] Engineering Technology Research Center for Building and Decorating Materials of Bamboo State Forestry Administration, China National Bamboo Research Center, Hangzhou 310012, China
[3] Zhejiang Academy of Forestry, Hangzhou 310023, China
[4] Luzhou Academy of Forestry, Luzhou 646000, China
* Correspondence: lvjing@cup.edu.cn (J.L.); gdk665@caf.ac.cn (D.G.)

Supporting Information

Includes:

Materials and methods

Figures S1–S9

Tables S1–S9

ANOVA Statistical Test of Mechanical Properties

One-way analysis of variance (ANOVA) was performed separately for the four mechanical properties: tensile modulus, tensile strength, flexural modulus, and flexural strength. First, the homogeneity of variances among groups was verified using Levene's test, confirming that the assumptions for ANOVA were met. Subsequently, F-values for each indicator were obtained by calculating the ratio of the mean square between groups (MSB) to the mean square within groups (MSW). The corresponding p-values for each indicator were then evaluated to determine whether they were less than 0.0001, thereby deciding whether to reject the null hypothesis.

Following this, Tukey HSD post hoc multiple comparison analysis was conducted to clarify specific intergroup differences. Additionally, the effect size η^2 was calculated to assess whether it exceeded 0.99, thereby evaluating the practical significance of the observed group differences.

ANOVA Statistical Test of WA and TS

Two-way ANOVA was conducted for the two variables of water absorption (WA) and tensile strength (TS). First, the homogeneity of variances across experimental groups was verified using Levene's test, confirming that the assumptions for the analysis were met. Subsequently, the corresponding F-values were obtained by comparing the ratio of the mean squares of each main effect and interaction effect to the mean square of the error. The statistical significance of the analysis results was then assessed.

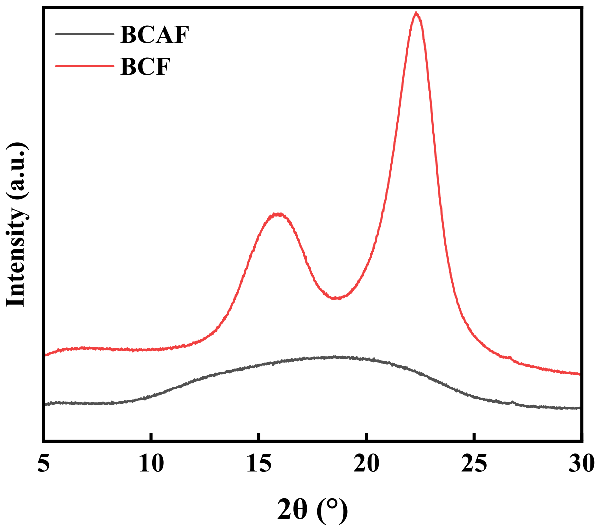

Figure S1. XRD of BCAF and BCF

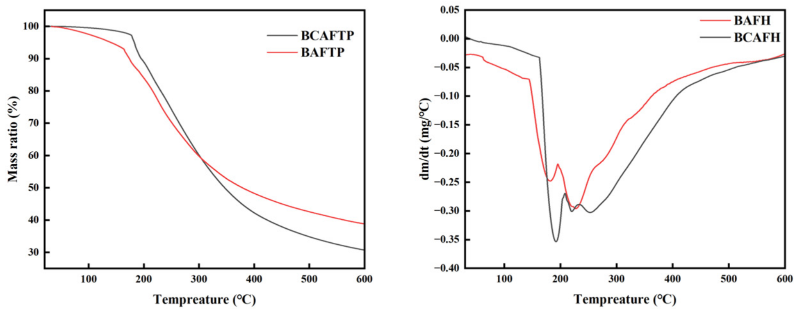

Figure S2. TGA and DTG of BCAFTP and BAFTP

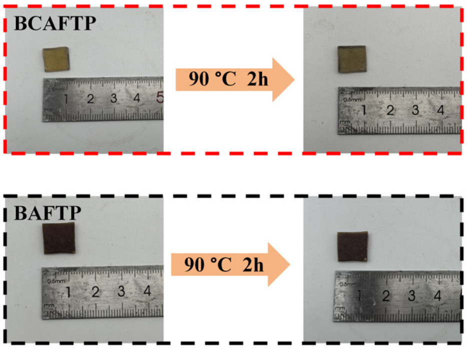

Figure S3. High temperature stability test (90 °C)

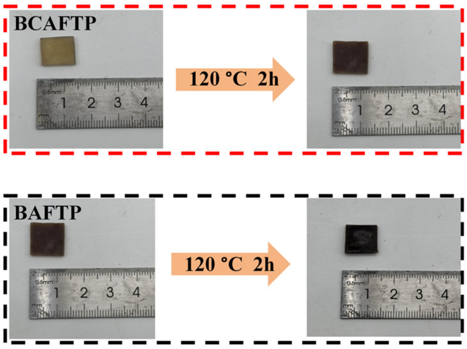

Figure S4. High temperature stability test (120 °C)

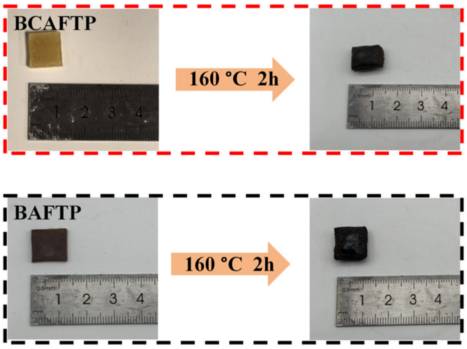

Figure S5. High temperature stability test (160 °C)

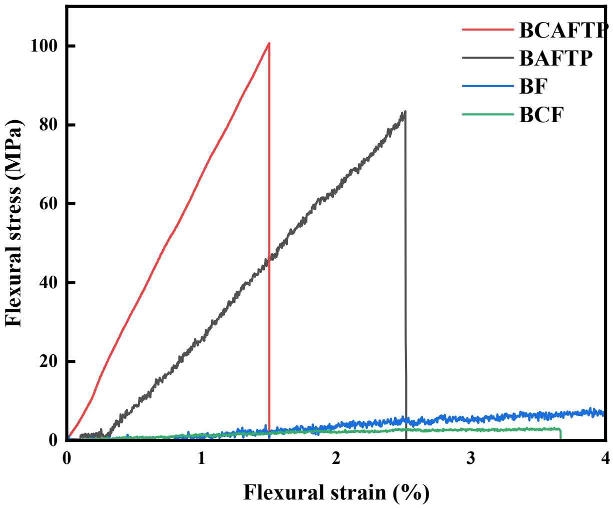

Figure S6. Comparison of flexural curves

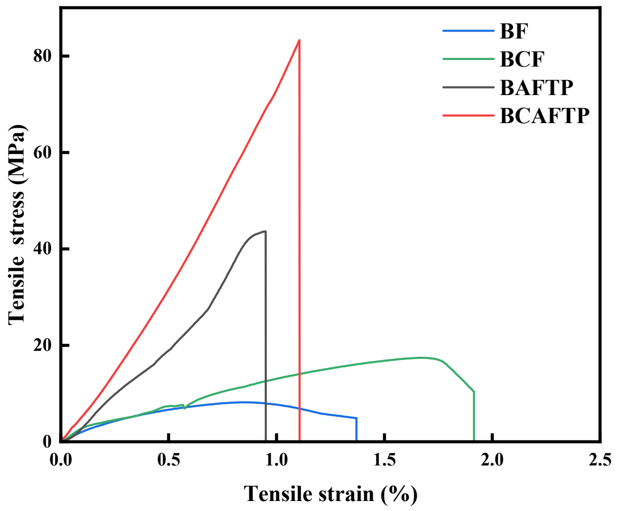

Figure S7. Comparison of tensile curves

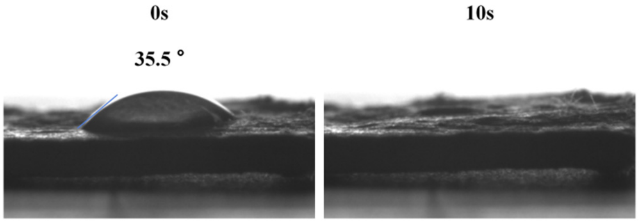

Figure S8. Contact angle of BF

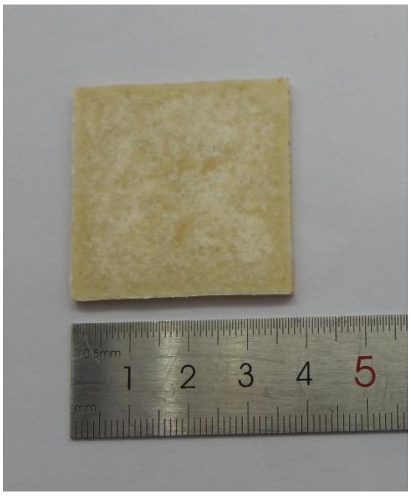

Figure S9. Inadequate hot pressing results in unformed samples

Table S1. Comparison of lignin content between BF and BCF

	BF	BCF
Lignin content (%)	1.67	0.99

Table S2. Statistical analysis of BF dimensions

BF	Length/ mm	Diameter/ μm
Average	1.25	52.27
Max	2.58	81.89
Min	0.55	38.16

Table S3. Statistical analysis of BCF dimensions

BCF	Length/ mm	Diameter/ μm
Average	1.33	52.76
Max	2.77	70.10
Min	0.59	33.72

Table S4. Statistical analysis of BAF dimensions

BAF	Length/ mm	Diameter/ μm
Average	1.39	22.75
Max	2.53	35.19
Min	0.52	17.56

Table S5. Statistical analysis of BCAF dimensions

BCAF	Length/ mm	Diameter/ μm
Average	1.47	20.02
Max	2.32	31.19
Min	0.51	14.06

Table S6. Single factor ANOVA analysis of flexural properties

Property	SSB	SSW	MSB	MSW	F-value	p-value(approx.)
Flexural Strength	60037.95	326.27	20012.65	11.6525	1717.47	$< 1\times10^{-18}$
Flexural Modulus	303.336	1.7927	101.112	0.064025	1579.28	$< 1\times10^{-18}$
Conclusion	The F-values of both indicators are extremely high, with corresponding p-values far below 0.001, indicating that there are highly significant differences among the four material groups in both flexural modulus and flexural strength.					

Table S7. Single factor ANOVA analysis of tensile properties

Property	SSB	SSW	MSB	MSW	F-value	p-value (approx.)
Tensile Modulus	231.9035	1.39	77.3012	0.086875	889.07	$< 1\times10^{-18}$
Tensile Strength	21204.465	206.96	7068.155	12.935	546.27	$< 1\times10^{-15}$
Conclusion	The F-values of both indicators are extremely high, with corresponding p-values far below 0.001, indicating that there are highly significant differences among the four material groups in both tensile modulus and tensile strength.					

Table S8. Tukey HSD multiple comparisons results of mechanical properties

Sample	Tensile Modulus (GPa)	Tensile Strength (MPa)	Flexural Modulus (GPa)	Flexural Strength (MPa)
BF	1.388^{a}	5.6^{a}	0.022^{a}	4.4^{a}
BCF	1.177^{a}	8.157^{a}	0.075^{a}	8.3^{a}
BAFTP	6.769^{b}	53.738^{b}	5.516^{b}	83.444^{b}
BCAFTP	9.032^{c}	83.31^{c}	6.769^{c}	100.727^{c}
Note	Within the same column, means sharing the same superscript letter are not significantly different ($P > 0.05$), while different letters indicate statistically significant differences ($P < 0.05$) based on Tukey's HSD test.			

Table S9. BTPs' two factor ANOVA analysis of WA and TS

Metric	Significance of the main effect of time	Significance of the main effect of material	Significance of time × material interaction effect
TS	Yes ($F=491.65$, $P<0.0001$)	Yes ($F=837.22$, $P<0.0001$)	Yes ($F=183.04$, $P<0.0001$)
WA	Yes ($F=63.27$, $P<0.0001$)	Yes ($F=68.59$, $P<0.0001$)	Yes ($F=21.03$, $P<0.0001$)
